# Supplementary material for: Predictors for carotid and femoral artery intima-media thickness in a non-diabetic sleep clinic cohort
Source: PLoS One. 2021 Jun 4;16(6):e0252569. doi: 10.1371/journal.pone.0252569 (PMC8177540; doi:10.1371/journal.pone.0252569)
Supplement: S1 File — (DOCX) [file pone.0252569.s001.docx]

*Tables S1 and S2 shows the main group and the RFM Sub-Group demographics (A), medical history (B) and blood test results (C).*

*N = group size;* HDL = High-density lipoprotein; BSL = blood sugar level.

**Table S1: Main Group demographics (A), medical history (B) and blood test results (C).**

**A**

|  | N | Frequency | Percentage |
| --- | --- | --- | --- |
| Gender (male) | 296 | 162 | 54.7 |
| Ethnicity (Caucasian) | 283 | 192 | 67.8 |

**B**

|  | N | Frequency | Percentage |
| --- | --- | --- | --- |
| Hypertension history | 294 | 124 | 42.2 |
| Hypertension medication | 294 | 123 | 41.8 |
| Hypercholesterolemia history | 290 | 132 | 45.5 |
| Hypercholesterolemia medication | 293 | 112 | 38.2 |
| Smoking history | 291 | 125 | 43 |

**C**

|  | N | Mean ± SD | Range |
| --- | --- | --- | --- |
| Total cholesterol (mmol/L) | 290 | 5.1 ± 1 | 3 – 7.9 |
| HDL (mmol/L) | 279 | 1.3 ± 0.3 | 0.7 – 2.7 |
| Total cholesterol:HDL ratio | 279 | 4.1 ± 1.1 | 1.8 – 8.7 |
| Triglycerides (mmol/L) | 288 | 1.5 ± 0.8 | 0.5 – 5.4 |
| BSL fasting (mmol/L) | 296 | 5.3 ± 0.6 | 3.3 – 6.9 |

**Table S2: RFM Sub-Group demographics (A), medical history (B) and blood test results (C).**

**A**

|  | N | Frequency | Percentage |
| --- | --- | --- | --- |
| Gender (male) | 157 | 101 | 64.3 |
| Ethnicity (Caucasian) | 152 | 106 | 69.7 |

**B**

|  | N | Frequency | Percentage |
| --- | --- | --- | --- |
| Hypertension history | 157 | 70 | 44.6 |
| Hypertension medication | 157 | 68 | 43.3 |
| Hypercholesterolemia history | 156 | 74 | 47.4 |
| Hypercholesterolemia medication | 157 | 61 | 38.9 |
| Smoking history | 157 | 68 | 43.3 |

**C**

|  | N | Mean ± SD | Range |
| --- | --- | --- | --- |
| Total cholesterol (mmol/L) | 155 | 5.0 ± 0.9 | 3.0 – 7.9 |
| HDL (mmol/L) | 150 | 1.2 ± 0.3 | 0.7 – 2.1 |
| Total cholesterol:HDL ratio | 150 | 4.2 ± 1.2 | 2.1 – 8.7 |
| Triglycerides (mmol/L) | 154 | 1.6 ± 0.8 | 0.5 – 5.4 |
| BSL fasting (mmol/L) | 157 | 5.3 ± 0.6 | 3.3 – 6.7 |

**Table S3: Comparison of patient characteristics by** **RFM Sub-Group status (included versus not included).**

Differences between those included and those not included in the RFM Sub-Group, tested by t-test (A), Mann-Whitney U-test (B) or χ^2^ test (C), where appropriate.

BMI = body mass index; WHR = Waist/Hip Ratio; HDL = High-density lipoprotein; BSL = blood sugar level; BP = blood pressure; CCA = common carotid artery; CFA = common femoral artery; AHI = apnoea/hypopnea index; RDI = respiratory disturbance index; AI = arousal index; ODI>3% = oxygen desaturation index SpO_2_ desaturation >3%; SpO_2_<90% (%TST) = %total sleep time with SpO_2_ <90%; *Lowest SpO_2_ NREM = Lowest SpO_2_ in non-rapid eye movement sleep; Lowest SpO_2_ REM = Lowest SpO_2_ in rapid eye movement sleep.*

FRS = Framingham risk score.

^+^ Indicates significant (p<0.05) differences.

**A**

| Variable | p-value |
| --- | --- |
| Age (years) | 0.6 |
| BMI (kg/m^2^) | 0.1 |
| Height (cm) | 0.001^+^ |
| Weight (kg) | 0.002^+^ |
| Neck circumference (cm) | <0.001^+^ |
| WHR | 0.05^+^ |
| Total cholesterol (mmol/L) | 0.008^+^ |
| HDL (mmol/L) | <0.001^+^ |
| Tot cholesterol:HDL | 0.1 |
| BSL fasting (mmol/L) | 0.9 |
| Systolic BP (mmHg) | 0.2 |

**B**

| Variable | p-value |
| --- | --- |
| CCA IMT (mm) | 0.7 |
| CFA IMT (mm) | 0.4 |
| AHI (events/hr) | <0.001^+^ |
| RDI (events/hr) | <0.001^+^ |
| AI (events/hr) | <0.001^+^ |
| ODI>3% (events/hr) | 0.02^+^ |
| SpO_2_<90% (% TST) | 0.2 |
| Lowest SpO_2_ NREM | 0.9 |
| Lowest SpO_2_ REM | 0.1 |

**C**

| Variable | p-value |
| --- | --- |
| Gender (male) | <0.001^+^ |
| Hypertension history | 0.4 |
| Hypercholesterolaemia history | 0.5 |
| Smoking history | 0.9 |
| Ethnicity (Caucasian) | 0.5 |
| Hypertension med | 0.6 |
| Hypercholesterolaemia med | 0.8 |
| FRS risk level (a.u) | 0.3 |
| AHI category | <0.001^+^ |

**Table S4:** **RFM Sub-Group IMT base models.**

Base models for log transformed CCA IMT (A) and CFA IMT (B) showing the independent, non-sleep predictor variables in the RFM Sub-Group.

*B = unstandardized beta coefficient; S.E. = standard error of B; R^2^ = coefficient of determination; BP = blood pressure.*

^+^ Indicates significant (p<0.05) differences.

**A ln(CCA IMT)**

| Variable | B | S.E. | p-value |
| --- | --- | --- | --- |
| (Constant) | -1.475 | 0.186 | <0.001^+^ |
| Age (per decade) | 0.088 | 0.014 | <0.001^+^ |
| Waist:hip ratio (a.u) | 0.135 | 0.148 | 0.4 |
| Systolic BP (mmHg) | 0.002 | 0.001 | 0.03^+^ |
| R^2^ = 0.268 | | | |

**B ln(CFA IMT)**

| Variable | B | S.E. | p-value |
| --- | --- | --- | --- |
| (Constant) | -1.431 | 0.137 | <0.001^+^ |
| Age (per decade) | 0.073 | 0.015 | <0.001^+^ |
| Weight (kg) | 0.003 | 0.001 | <0.001^+^ |
| Total cholesterol:HDL ratio | 0.022 | 0.013 | 0.09 |
| R^2^ = 0.194 | | | |

**Table S5:** **[SDB variable x study group] interaction models for IMT.**

Parameter estimates for the interaction term [SDB variable x study group] when added individually to the base model, i.e. base model + SDB variable + study group + [SDB variable x study group] interaction, for log transformed CCA IMT (A) and CFA IMT (B).

*B* = unstandardized beta coefficient adjusted for base model; S.E. = standard error of B*, p-value* = p-value adjusted for base model; AHI = apnea/hypopnea index; RDI = respiratory disturbance index; AI = arousal index; ODI>3% = oxygen desaturation index SpO_2_ desaturation >3%;* SpO_2_<90% (%TST) = %total sleep time with SpO_2_ <90%*; Lowest SpO2 NREM = Lowest SpO_2_ in non-rapid eye movement sleep; Lowest SpO_2_ REM = Lowest SpO_2_ in rapid eye movement sleep.*

^+^ Indicates significant (p<0.05) differences.

**A ln(CCA IMT)**

| Variable | B* | S.E.* | p-value* |
| --- | --- | --- | --- |
| ln(AHI)*study group | 0.015 | 0.019 | 0.4 |
| ln(RDI)*study group | 0.038 | 0.028 | 0.2 |
| ln(AI)*study group | 0.066 | 0.043 | 0.1 |
| ln(ODI>3%)*study group | 0.000 | 0.020 | >0.9 |
| ln(SpO2<90%)*study group | -0.008 | 0.021 | 0.7 |
| ln (Lowest SpO_2_ NREM)*study group | 0.050 | 0.277 | 0.9 |
| ln (Lowest SpO_2_ REM)*study group | 0.246 | 0.178 | 0.2 |
| AHI >30 events/hr*study group | 0.010 | 0.068 | 0.9 |

**B ln(CFA IMT)**

| Variable | B* | S.E.* | p-value* |
| --- | --- | --- | --- |
| ln(AHI)*study group | -0.052 | 0.020 | 0.01^+^ |
| ln(RDI)*study group | -0.062 | 0.029 | 0.03^+^ |
| ln(AI)*study group | -0.055 | 0.047 | 0.2 |
| ln(ODI>3%)*study group | -0.050 | 0.021 | 0.02^+^ |
| ln(SpO_2_<90%)*study group | -0.052 | 0.021 | 0.01^+^ |
| ln (Lowest SpO_2_ NREM)*study group | 0.493 | 0.312 | 0.1 |
| ln (Lowest SpO_2_ REM)*study group | 0.397 | 0.192 | 0.04^+^ |
| AHI >30 events/hr*study group | 0.118 | 0.077 | 0.1 |

**Table S6: RFM Sub-Group SDB variable models for IMT.**

Results of adding each SDB variable individually to the base model, i.e. base model + SDB variable, for ln(CFA IMT) in the RFM Sub-Group.

*B* = unstandardized beta coefficient adjusted for base model; S.E. = standard error of B*, p-value* = p-value adjusted for base model; AHI = apnea/hypopnea index; RDI = respiratory disturbance index; AI = arousal index;*  ODI>3% = oxygen desaturation index SpO_2_ desaturation >3%; SpO_2_<90% (%TST) = %total sleep time with SpO_2_ <90%;  *Lowest SpO_2_ NREM = Lowest SpO_2_ in non-rapid eye movement sleep; Lowest SpO_2_ REM = Lowest SpO2 in rapid eye movement sleep; snores/hr = snores per hour, Δ**R^2^ is the incremental change in R^2^ after addition of the SDB variable to the Base Model calculated as (SDB model R^2^- Base Model R^2^).*

*^+^ Indicates significant (p<0.05) differences.*

| Variable | B^*^ | S.E.^*^ | p-value* | ΔR^2^ |
| --- | --- | --- | --- | --- |
| ln(AHI) | 0.034 | 0.014 | 0.01^+^ | 0.033 |
| ln (RDI) | 0.057 | 0.022 | 0.01^+^ | 0.037 |
| ln (AI) | 0.062 | 0.030 | 0.04^+^ | 0.001 |
| ln (ODI>3%) | 0.016 | 0.012 | 0.2 | 0.010 |
| ln (SpO_2_<90%) | 0.010 | 0.013 | 0.5 | 0.003 |
| ln (Lowest SpO_2_ NREM) | -0.182 | 0.176 | 0.3 | 0.006 |
| ln (Lowest SpO_2_ REM) | -0.102 | 0.095 | 0.3 | 0.028 |
| ln (Snores/hr) | -0.020 | 0.022 | 0.4 | -0.017 |
