# Supplementary material for: Predictors for carotid and femoral artery intima-media thickness in a non-diabetic sleep clinic cohort
Source: PLoS One. 2021 Jun 4;16(6):e0252569. doi: 10.1371/journal.pone.0252569 (PMC8177540; doi:10.1371/journal.pone.0252569)
Supplement: S1 Protocol — (PDF) [file pone.0252569.s002.pdf]

SWAHS (Westmead Campus) Human
Research Ethics Committee

APPROVED

Date: 25.5.10

INTRODUCTION:

Snoring and mild sleep disordered breathing (SDB) are a common consequence of increased upper airway resistance during sleep (3). Habitual snoring (every night or almost every night) without overt obstructive sleep apnoea hypopnoea syndrome (OSAHS) is highly prevalent in the community, occurring in approximately 40% of men and 20% of women (5, 60). Increasingly, it is recognized that snoring and mild SDB are not just an annoying social problem (24) but may pose real risks to health. Emerging data are highly suggestive of an independent role in the pathogenesis of hypertension (10), stroke (31), coronary (35) and carotid atherosclerosis (JW15).

Our research proposal focuses on the relationship between heavy snoring with mild SDB and early changes of carotid artery atherosclerosis and is supported by results from our previous study identifying heavy snoring as a risk factor for carotid atherosclerosis (JW15), and our animal model studies confirming transmission of snoring vibration energy to carotid artery walls (JW9, JW12).

The aim of this study is to further explore the linkage between heavy snoring with mild SDB and early carotid artery atherosclerosis, by demonstrating regression of early changes of carotid atherosclerosis in heavy snorers with mild non-hypoxic SDB, when snoring and SDB are prevented by the use of nasal continuous positive airway pressure (CPAP). We will achieve this aim by testing for carotid atherosclerosis regression and/or stabilisation with a prospective, randomised controlled trial of nasal CPAP in heavy snorers with mild to moderate SDB.

We will test the following hypothesis:
Early changes of carotid artery atherosclerosis present in patients with heavy snoring and mild SDB will stabilise or regress following treatment with nasal CPAP therapy.

BACKGROUND:

Snoring sounds are generated in the upper airway during sleep and result from vibration of the pharyngeal wall and its associated structures (37). Over a decade ago, Hedner et al. (23) proposed that snoring vibrations may be transmitted through surrounding tissues to the carotid artery wall. They hypothesised that, repeated nightly over time, these vibrations may result in: 1) initiation/promotion of carotid artery endothelial damage, a recognised pathogenic factor for atherosclerosis; and/or 2) promotion of existing plaque rupture, leading to thrombus dislodgement and embolic stroke. The carotid artery lies in close proximity to the pharyngeal wall, and the juxtaposition of the pharynx and the carotid bifurcation, the major site of atherosclerotic lesions in the carotid artery (62), adds to the plausibility of this scenario. In this paradigm, snoring vibrations represent a potential energy source for both the initiating (intimal injury) and terminating (plaque rupture) events of the "reaction to injury" cascade theory (52) for carotid atherosclerosis.

OSAHS is amongst the commonest chronic disorders of adults, with a prevalence in the order of 4% for middle aged men and 2% for middle aged women (60). OSAHS is now generally regarded as an independent risk factor for hypertension, myocardial infarction and stroke (2, 43, 61). Recently, two studies in patients with untreated polysomnographically (PSG) verified severe OSAHS (16, 46), who were at low risk from the classical risk factors for cardiovascular diseases, have demonstrated the presence of early carotid atherosclerosis (increase in intima-media thickness [IMT]). Furthermore, when a group of severe OSAHS subjects with severe nocturnal hypoxia were treated with CPAP for 4 months, there was a decrease in carotid IMT (17). Snoring was not measured in these studies but given its strong association with OSAHS, snoring was very likely to have been present. Thus it has been demonstrated that patients with severe OSAHS accompanied by nocturnal hypoxia may develop early changes of carotid atherosclerosis, and that these changes can regress following prevention of OSAHS and hypoxia by the use of nasal CPAP.

Snoring has been identified as a risk factor for chronic cardiovascular diseases including hypertension (44, 61), myocardial infarction (14), ischemic heart disease (31), and stroke (25, 48, 54). Three cohort studies have demonstrated an increased relative risk of stroke in habitual snorers

with odds ratios of 1.26 - 2.13 (25, 27, 31). However, the relative role of snoring and OSAHS in these studies is unclear since snoring and OSAHS frequently co-exist. All the above studies suffer from the limitation that the information regarding snoring is derived from questionnaires rather than objective measurements. In addition, these studies are also confounded by the presence of OSAHS: indeed, most authors have used snoring as a surrogate marker for OSAHS.

The pathophysiological mechanisms that link snoring, OSAHS, and carotid atherosclerosis are not clear, and remain largely unexplored. Recently intermittent hypoxia has been linked with atherosclerosis through pathways leading to endothelial dysfunction (34). In support of this, studies of OSAHS patients have demonstrated a relationship between carotid IMT and nocturnal hypoxia (16, 56). However, nocturnal hypoxia is not invariably a feature of OSAHS, whereas snoring is nearly always present. Thus, although severe, hypoxic OSAHS has been independently associated with carotid atherosclerosis and stroke, the mechanisms underpinning this linkage remain unclear. Indeed, until recently, the association between heavy snoring with mild SDB and carotid atherosclerosis had not been investigated.

We recently published a large cross-sectional study (JW15) in 110 snorers of varying severity (mild to no OSAHS), with adequate power to assess associations between snoring and carotid vascular disease. Subjects underwent nocturnal PSG with objective snore measurement and quantification using a microphone. Ultrasound examination of both carotid and femoral arteries was performed. Cardiovascular risk factors were assessed, including age, gender, body habitus, hypertension, lipid status and smoking history. Measured sleep-disordered breathing (SDB) variables included the apnoea-hypopnoea index (AHI), arousal index (AI), indices of nocturnal hypoxia, and snoring (percent sleep time snoring). Logistic regression was used to determine predictors for the presence of carotid and femoral atherosclerotic plaque. The prevalence of carotid atherosclerotic plaque was 31%, and femoral atherosclerotic plaque 22%. Significant risk factors for carotid atherosclerotic plaque were age, male gender, hypertension, smoking history and **heavy snoring**. However, the AHI, AI and nocturnal hypoxia were **not** significant risk factors.

The adjusted odds ratio for carotid atherosclerosis in the heavy snoring group (snoring greater than 50% sleep time) was substantially increased at 10.5 (2.1 – 51.8, 95% CI), and was clearly the strongest association with carotid atherosclerosis, with odds ratios much higher than any of the traditionally accepted risk factors for atherosclerosis (odds ratios 3.2 to 4.7). Since this was not a community based population study, the strength of the odds ratio for heavy snoring likely reflects the specific group chosen for study, consisting of heavy snorers with mild SDB, minimal hypoxia, and a high prevalence of background hyperlipidaemia and hypertension. Thus we do not consider that the strength of the odds ratio indicates the general population risk for developing carotid atherosclerosis in relation to heavy snoring. Nevertheless, the results confirm a strong snoring effect with carotid atherosclerosis risk once there is adjustment for traditional risk factors. In contrast, significant associations for femoral atherosclerosis were limited to the more traditional risk factors of age, gender and smoking history, with no significant association with any measure of SDB. From these data we concluded that heavy snoring was a specific risk factor for carotid atherosclerotic plaque development. Our study is the first to demonstrate that snoring per se (separate from OSAHS and accompanying nocturnal hypoxia) is a strong independent risk factor for carotid atherosclerosis. In support of this, the prevalence of carotid plaque in our population of heavy snorers was more than 1.8 times greater than that reported for the general population (26), and even higher than that seen in OSAHS populations (46).

The novelty and importance of this study were recognised by the publication of two separate editorial commentaries in the journal *Sleep* (18, 42), both of which concluded that the relationship between snoring and carotid atherosclerosis is an exciting new area of research. They noted that the significance of the finding may elevate the importance of snoring from a social annoyance to a vascular risk factor. If the associations observed in this study are indeed causal, the significantly

raised risk of carotid atherosclerosis in heavy snorers with mild SDB, coupled with the high prevalence of snoring and mild SDB in the community, has substantial public health implications for the prevention of stroke. However, the conclusion so far is that there is an independent association between snoring and carotid atherosclerosis. Both editorial commentaries concluded that many important investigative questions still remain, and recommended that future treatment studies focusing on patients with heavy snoring and mild SDB should be performed in order to clarify the relationship between snoring and atherosclerosis of the carotid arteries by assessing the impact of treatment of heavy snoring with an appropriate treatment regime.

Physiological Changes related to Snoring and mild SDB that may lead to Carotid Artery Atherosclerosis

Carotid atherosclerosis is complex disease involving several highly inter-related processes including endothelial dysfunction, inflammation, oxidative stress, vascular smooth muscle cell activation, platelet activation and thrombosis (52, 59). Ultimately, atherosclerosis is an inflammatory disease of large and medium-sized arteries in which vascular endothelial injury or dysfunction leads to an advanced, complicated lesion due to chronic inflammatory processes. Not only do the earliest changes of atherosclerotic lesions occur in the endothelium, but normal endothelium also has a crucial role in the prevention of the development of atherosclerosis (59).

OSAHS is characterised by chronic intermittent hypoxia (CIH) in a significant number of the more severe cases. Recently, much attention has been focused on the role of nocturnal CIH as an atherogenic factor linking OSAHS and vascular disease (22, 34). Studies have shown independent associations between hypoxic stress of OSAHS and increased carotid artery IMT (16). However, there was a lack of relationship between nocturnal hypoxia and carotid atherosclerosis as observed in our study (JW15), demonstrating that nocturnal CIH is clearly not a prerequisite feature for developing carotid atherosclerosis. Thus the pathophysiologic changes that link snoring, OSAHS and carotid atherosclerosis are not clear, and mechanisms other than nocturnal CIH need to be considered.

Atherosclerosis tends to develop in regions of the circulation that are subject to low or turbulent flows such as at bifurcations of arteries (21). It is well established that areas of low or non-laminar shear stress (the tangential force of blood over the endothelium) are prone to atherogenesis by changing the normally quiescent endothelial phenotype to that of an aggressive, pro-inflammatory phenotype (41). Thus, maintaining normal wall shear stresses is critical to preventing increased susceptibility to atherogenesis (12).

Reductions in intra-pleural pressure are known to lower cardiac output via an increase in left ventricular afterload due to increased systolic left ventricular transmural pressure with resultant reduction in left ventricular filling and greater right ventricular filling (ventricular interdependence) with impaired left ventricular relaxation (8). Since snoring is associated with increased upper airway resistance and reductions in peak inspiratory pleural pressure (55), associated reductions in cardiac output and, therefore, carotid artery blood flow, appear likely.

The carotid arteries are extremely close to the pharyngeal wall (within 10-20mm) and the carotid bifurcation (the major site of atherosclerotic plaques) is directly adjacent to the pharyngeal segment of the upper airway. During snoring, a high energy ~ 60 Hz oscillatory pressure wave is generated within upper airway tissues (38), including the soft palate, pharyngeal wall, epiglottis and tongue (37). Recently, we demonstrated that induced snoring in an animal model resulted in pressure vibrations in both the tissues surrounding the carotid artery wall and the artery lumen (JW9).

The local effects of vibration of the carotid artery walls during snoring may also contribute to changes in blood flow velocity and/or vessel diameter. Acute application of a continuous vibration stimulus to the human external iliac artery resulted in dilatation over 7-10 hours (7). Intermittent

one minute vibrations to both an intact and denervated canine forelimb resulted in vasodilatation at all levels of vibration (36). Acute vibration of the rat tail artery over four hours resulted in an enhanced vasoconstriction response to α–adrenergic antagonists (32). Thus, it is clear that acute vibration of arterial walls can result in changes in arterial diameter and vessel responsiveness, which may result in changes in blood flow velocity and wall shear stress, although this effect has never been characterised for the carotid artery.

On a more chronic basis it is thought that mechanical vibrations imposed on arterial walls cause alterations in wall elastin and vascular smooth muscle which in the long term lead to arterial dilatation (7, 15). Changes in arterial diameter over time will result in changes in carotid artery blood flow velocity, bulk flow and flow turbulence. In addition, repetitive changes in blood flow velocity will increase mechanical stress on the carotid artery wall. Mechanical stresses on the arterial wall have been shown to participate in the pathogenesis of atherosclerosis as local factors (28, 29).

Snoring vibration transfer to carotid artery walls also provides a potential source of trauma to carotid arterial wall structures including the vascular endothelium. The concept of vibration-mediated damage to blood vessels is well established for other peripheral arteries in the body (30). Recent data from studies in rats also supports the potential of vibratory stimuli to cause arterial endothelial damage. Intermittent vibration (for between 4 hours and 9 days) of rat tails at 60 Hz (a frequency similar to human snoring) results in injury to the tail artery with initial vasoconstriction, injury of endothelial cells, and subsequent endothelial denudation (13). Indeed, this study demonstrated that endothelial cells showed signs of injury after a single four-hour bout of vibration. Electron microscopy revealed vacuolation with loss and thinning of endothelial cells, with activated platelets coating the endothelium. Endothelial and vascular smooth muscle cells contained double membrane-limited, swollen processes indicative of vaso-constriction induced damage (13). This progressed to extensive endothelial cell death after nine days of vibration. Notably, the carotid artery is more sensitive to injury than other peripheral arteries (51). Therefore, even a small repetitive mechanical injury to the carotid artery may result in endothelial damage.

The potential for snoring vibrations to injure tissues is evidenced by the pathology described in the upper airway tissues (oedema, nerve fibre degeneration) of habitual snorers (19, 58). In addition, snoring-like vibration of human bronchial cell culture (50) and the rat upper airway (1) induces a proinflammatory response.

Given the above background, the concept that snoring and mild SDB may damage the carotid artery endothelium seems entirely plausible. For this application, the only mechanism that will not be treated is that of hypoxia, as the study population will be chosen on the basis of an absence of nocturnal hypoxia as part of their clinical phenotype. However, as CPAP therapy will effectively treat most of the mechanisms related to snoring and SDB that may lead to carotid atherosclerosis, this study is not designed to determine which of these factors is the major cause of injury to the carotid artery. CPAP will eliminate snoring vibrations, reductions in pleural pressure, changes in carotid artery blood flow and wall stress, and direct vibration trauma to the arterial endothelium. Any of these mechanisms may be responsible for endothelial dysfunction developing during snoring and SDB, so the proposed trial will not be able to determine the physiological cause of any clinical response to CPAP treatment that is observed as an outcome. Indeed, we have a currently funded NH&MRC project grant entitled *'The role of snoring vibrations in the pathogenesis of early carotid artery atherosclerosis'*, that contains a series of studies that will help to determine the mechanisms by which snoring may lead to carotid atherosclerosis and endothelial dysfunction, using both animal and human models to separate the various physiological mechanisms. Preliminary results from an animal study demonstrate that direct mechanical vibration of the carotid artery (with snoring-like vibratory energy) reduces both baseline and acetylcholine-induced carotid artery cGMP by >40%, suggesting decreased endothelial nitric oxide bioavailability or endothelial dysfunction, a known

precursor to atherogenesis. This supports the potential for snoring vibration energy to directly initiate carotid artery endothelial damage. This previously funded study does not contain any clinical trial or trial of therapy. The current proposed study is entirely different, as it is a large clinical trial which proposes to study whether there is any clinical benefit to subjects with heavy snoring and mild SDB from treatment with CPAP in terms of prevention and regression of carotid artery atherosclerosis. We do not propose to study the changes in vascular physiology that may lead to any clinical benefit.

Quantification of Carotid Artery Atherosclerosis with Intima-Media Thickness (IMT)

In our recent cross-sectional study (JW15), carotid atherosclerosis was graded based on the presence or absence of plaque in the common or internal carotid arteries using ultrasound detection (JW15). Atherosclerosis was graded as mild if the plaque caused an estimated artery stenosis of less than 50%. This method of grading does not use a linear scale, so will be less suitable for measuring regression of atherosclerosis over 12 months, as is proposed in this study. Instead, carotid IMT will be used as it is an intermediate phenotype for early atherosclerosis, and is quantifiable on a linear scale. It will serve as the primary outcome variable for this project. Because it can be measured relatively simply and non-invasively, it is well suited to larger clinical studies. Ultrasonic measurements correlate well with histology (49), and increased IMT is associated with vascular risk factors (6, 9, 53) and the presence of more advanced atherosclerosis (47). Increasingly, IMT is being used to stratify risk and as an end point in clinical intervention studies. Its use relies on its ability to predict future clinical cardiovascular end points. A systematic review and meta-analysis of relevant studies and their data demonstrated that carotid IMT is a strong predictor of future vascular events (39), with an adjusted relative risk of stroke of 1.18 (95% CI, 1.16 to 1.21) per 0.10 mm common carotid artery IMT increase. MRI is also a promising new technology that can measure carotid artery Mean Wall Thickness (MWT), which is strongly correlated with carotid ultrasound IMT measurements (11). However, this is still a relatively new technique which does not have established methodology, has not been shown to be predictive of future vascular events, and is relatively expensive. For these reasons, ultrasound IMT remains the gold standard for detecting early changes of carotid atherosclerosis, and will be used in this project.

Our quantification of IMT will follow recently published standardised methodology for its measurement (57). Previous reports have demonstrated that replicate measures of IMT have an average absolute difference of between 0.06 and 0.20mm (39). Our power calculation incorporates this degree of test repeatability in calculating our sample size. Ultrasound studies will all be performed in the Ultrasound Department at Westmead Hospital by two experienced senior sonographers under the direction of Dr George Larcos (Head of Clinical Department), which will minimise between-technician variations. The technicians and reporting physician will be blinded as to the treatment status of patients undergoing carotid ultrasound tests. Equipment time for one study per day is available in our Ultrasound Department, and studies will generally be performed early or late in the day, either before or after routine clinical studies scheduled in the Department.

The potential for atherosclerosis regression or stabilisation with removal of an atherogenic stimulus has been confirmed in several studies. For example, there are now several clinical studies that have demonstrated reductions in carotid IMT (indicative of regression of early atherosclerosis) over a six to twelve month period with the lipid profile altering medications Pravastatin and Lovastatin (20, 40, 45). These studies have consistently demonstrated significant regression or slowing of progression of carotid IMT with therapy, and benefits of therapy were evident after as little as six to twelve months (45). If heavy snoring with mild SDB (non-hypoxic) constitutes an atherogenic stimulus, it therefore seems plausible that the removal of this stimulus by treatment with nasal CPAP therapy may well be associated with regression or stabilisation of the early changes of atherosclerosis within a six to twelve month time frame. In support of this, a recent study by Drager et al (17) demonstrated that effective treatment of a group of 12 severe, hypoxic

OSAHS patients with CPAP for 4 months resulted in a significant decrease in carotid IMT of 0.063mm, compared with a small increase of 0.008mm in 12 control OSAHS subjects. All subjects were selected to be at low risk of confounding cardiovascular risk factors. Overall, this represented a 9% reduction in IMT over only a four month treatment period. We believe that this study provides strong support that CPAP treatment is able to cause significant reductions in carotid IMT within a reasonable timeframe that makes our hypothesis a testable proposition that would help to establish benefits of CPAP treatment in a group of patients with heavy snoring and mild SDB.

Cerebral microvascular disease can now be readily quantified by use of digital retinal photography, and the qualitative and quantitative analysis of the retinal microvasculature has emerged as a powerful non-invasive tool for assessment of the microcirculation. Retinal photography has been revolutionised by advances in imaging and computer techniques that permit retinal microvascular signs to be non-invasively studied in great detail with computerised image processing techniques. High quality digital photographs of the retina can be obtained using a non-mydriatic retinal camera. In recent years, reliable assessment methods have been developed and refined leading to a battery of qualitative and quantitative methods for evaluating retinal signs.

Quantitative assessment of retinal vascular signs includes estimates of the internal calibre of retinal arterioles, venules and the ratio of the two: the arteriole-to-venule ratio (AVR). Recent large population-based studies have demonstrated that relative narrowing of retinal arteriolar calibre or relative widening of retinal venular calibre carry predictive and prognostic significance for some systemic conditions including stroke.

RESEARCH PLAN:

PROJECT: Regression of Carotid Atherosclerosis in Patients with Heavy Snoring and mild SDB using CPAP Therapy.
Hypothesis: Early changes of carotid artery atherosclerosis present in patients with heavy snoring and mild SDB will stabilise or regress following treatment with nasal CPAP therapy.

Design: *This will be a prospective, randomised, controlled study* of 130 patients aged between 45 and 70 years, diagnosed with heavy snoring (snoring > 50 percent total sleep time), but only mild to moderate, non hypoxic OSAHS (AHI < 30 events/hour, oxygen desaturation index 3% [ODI] < 5 events/hour). Subjects with habitual snoring will be reviewed in our sleep clinic. Previously, we recruited a similar cohort of 110 heavy snorers in our study of snoring as a risk factor for carotid atherosclerosis (JW15). We anticipate no difficulty in recruiting a new cohort of subjects from similar sources. The majority of patients screened will come from the cohort of patients undergoing routine diagnostic PSG studies for assessment of SDB.

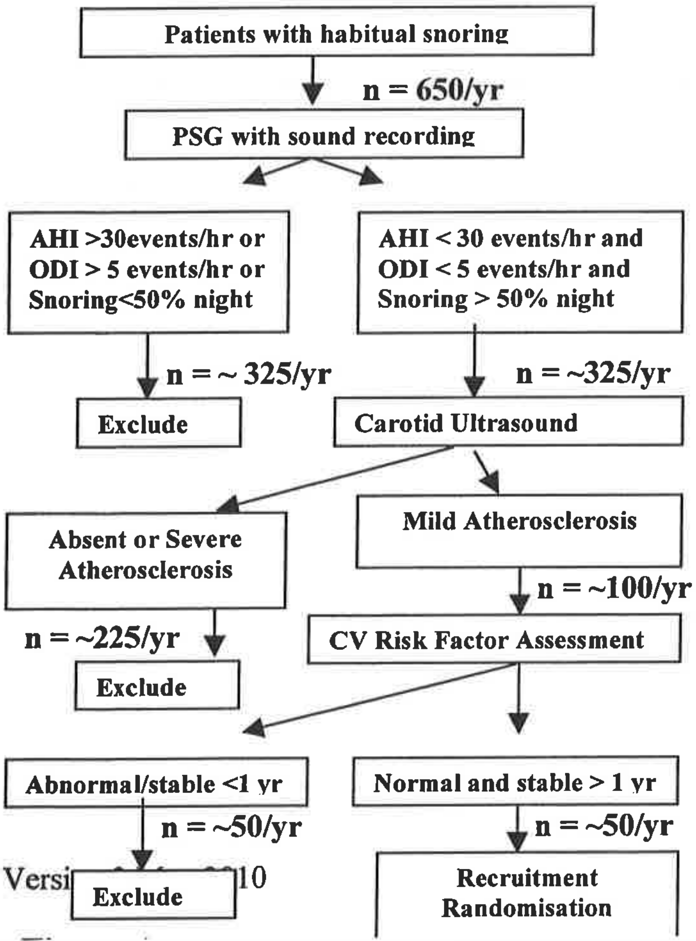

Screening Protocol (see Figure 1 and Table 1):
Subjects will initially be screened for eligibility to enroll:

i) Prospective subjects will be identified by a history of habitual snoring. Our laboratory performs more than 650 diagnostic PSG studies in potentially suitable subjects each year. Following a full clinical history, prospective subjects will be excluded if there is a history of previous stroke, diabetes, severe nasal disease, current or recent smoking during the past year, carotid artery surgery or inability to tolerate nasal CPAP therapy.

ii) *Standard PSG together with snore sound recording* will then be performed to correctly categorise patients into the heavy snoring group. Snoring will be quantified by manual counting of snoring epochs from the PSG, and subjects who snore > 50% of total sleep time, with an AHI < 30 events/hour and ODI 3% < 5 events/hour (JW15), will proceed to the next screening step. Approximately 50% of our sleep laboratory subjects met these criteria in 2008.

iii) *Carotid artery ultrasound* with colour coded Doppler will be performed on the right and left internal and common carotid arteries in patients with mild to moderate SDB with heavy snoring. Atherosclerosis (presence of plaque) and IMT will be quantified, and subjects excluded if there is no atherosclerosis, or severe carotid stenosis (> 70% narrowing) when they will be referred for surgical assessment. Subjects with mild to moderate atherosclerosis (presence of any plaque with estimated carotid stenosis degree of 1-70%; [JW15]) will continue in the screening protocol.

iv) *Retinal photography*- we will take retinal photographs before (pm) and after (am) sleep to quantify retinal microvascular lesions including retinal emboli.

v) *Based on our previous study* (JW15), approximately 30% of subjects will have at least mild to moderate carotid atherosclerosis on ultrasound. We will then undertake a *cardiovascular risk factor assessment* (review of anthropometry, lipid status, blood pressure, smoking history, and blood sugar) for stability over the past year. Stability will be defined by blood tests and history as: no current abnormal risk factors with no history of risk factor treatment or abnormality over the prior year; or history of abnormal risk factors with stable medical therapy for greater than one year which normalises the risk factors historically and currently. Stable subjects with normal blood pressure (<140/90 mmHg), normal fasting blood sugar level and blood lipid profile, and who are current non-smokers will be eligible to be enrolled. We have allowed for a 50% ineligibility rate at this step.

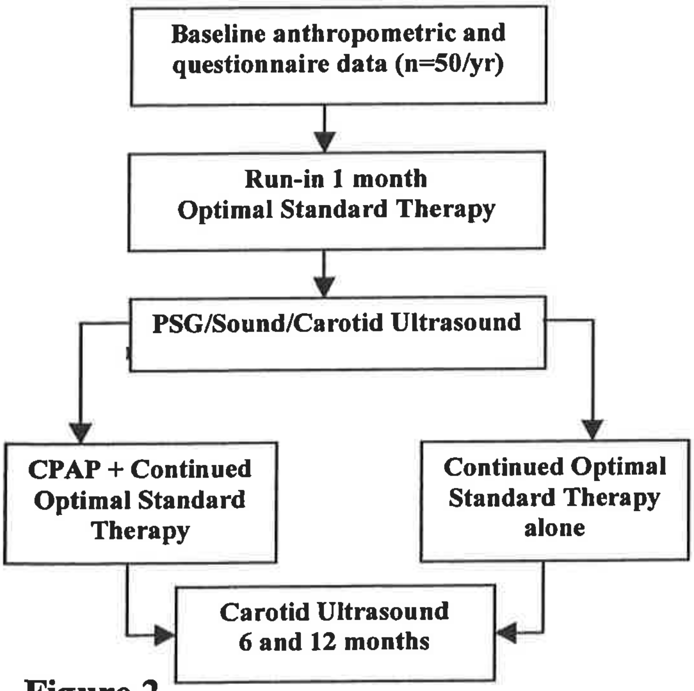

Figure 2

Study Protocol (see Figure 2 and Table 1):

i) *We will record anthropometric data*, cardiovascular risk factors, clinical examination, and an in-house quantifiable Westmead Snore History Questionnaire (WSHQ).

ii) *Recruited subjects (n=50/year) will complete a 1 month run-in period*, with review for stability of clinical history, blood lipids, and blood pressure designed to ensure that subjects are on optimal standard therapy for cardiovascular risk factors (normal values with no clinically significant changes). *Optimal standard therapy* will include continued non-smoking, maintenance of weight, medication to keep blood pressure and blood lipid profile normal. At the end of this period, diagnostic PSG with calibrated sound recording and neck accelerometer recording (to measure vibration energy) will be undertaken. Repeat carotid artery Doppler ultrasound will be performed prior to randomisation.

iii) *Following the 1 month run-in period, subjects will be randomised* to either a further 12 month period of continued optimal standard therapy, or optimal standard therapy plus nightly nasal CPAP treatment. Randomisation will be stratified according to medication use for cardiovascular risk factors at baseline, with randomisation in blocks of 4 into each treatment arm. Subjects in the CPAP arm will have a laboratory CPAP titration study, to eliminate snoring and obstructive events. The study treatment period will run for 12 months, with monthly monitoring of CPAP compliance (machine hours at pressure recorded), and continued treatment of cardiovascular risk factors.
iv) *Repeat carotid artery Doppler ultrasound examinations* will be performed at six months and at the conclusion of the treatment period together with measurement of cardiovascular risk factors and repeat questionnaires. Carotid artery ultrasound will measure IMT, plaque type and extent, carotid diameter, and peak systolic velocity. An increase in IMT of >0.03 mm/year is detectable and clinically relevant, as it is associated with a doubling in incidence of coronary events (47).

TABLE 1: Schedule of Visits and Required Procedures

VISIT	Screen		Run-In	Treatment Period				
Week of Study	-7	-5	-1	0	4-24	26	30-50	52
Visit Number	1	2	3	4	5-10	11	12-17	18
History & Examination	X		X	X		X		X
Anthropometry	X		X	X		X		X
PSG / Sound Recording	X		X					
Carotid Ultrasound		X	X			X		X
CV Risk Factor Assess		X	X	X	X	X	X	X
Blood - BSL, lipids, CRP		X	X			X		X
Eligibility Assessment		X						
WSHQ	X		X			X		X
Randomisation			X					
CPAP Titration PSG				X				
Adverse Events			X	X	X	X	X	X
Telephone Contact					X		X	
CPAP Compliance					X	X	X	X
Medication Compliance		X	X	X	X	X	X	X

We have chosen not to have a placebo in the control arm of this study due to the well recognized problems with appropriate controls for CPAP therapy. 'Sham CPAP' provides a minimum pressure of 4 cmH_2O which may have a confounding therapeutic effect in our trial and hence is not suitable (and it will also be difficult to maintain patient compliance over this time period). A placebo tablet would have to be presented to the patient as having a possible therapeutic effect on carotid atherosclerosis, which would not be ethically acceptable. Treatment with an active placebo may confound our ability to detect a CPAP response, or would require three arms to the trial, significantly increasing the study population required and the overall cost of the trial. In any case, our primary outcome variable will be change in carotid IMT which is an objective measure, unlikely to be affected by placebo response, thus not requiring a placebo-treated control group.

We acknowledge that compliance with CPAP treatment will be a critical factor to the success of this trial. Patients will be offered as much support as required in the initial phases of the study (both by regular telephone contact and visits as required) to ensure that technical and comfort problems are dealt with optimally. To maximize compliance, each patient on CPAP will be reviewed in person two weeks after starting on treatment, and then telephoned every four weeks to discuss and resolve problems and to encourage nightly use of the treatment. They will be able to contact the study co-ordinator at any time to resolve problems, and additional visits will be expedited where

required. CPAP compliance will be monitored objectively by nights and hours of use from machine download at study visits, and also qualitatively by questionnaire at each telephone contact. Compliance with routine medication use will similarly be qualitatively assessed at the same time points. Overall objective compliance with CPAP was 6 hours per night in the Drager study (17), and between 3.5 and 4.4 hours per night in two studies in patients with mild to moderate OSAHS (4, 33), similar in design to this study. We will aim to target a minimum compliance of 4 hours per night, and analysis of results will be stratified based on compliance measures.

We also acknowledge that maintaining subject involvement in this study will be challenging, and that there may well be a significant drop out rate, particularly in the CPAP arm. Previous studies using CPAP to treat mild to moderate OSAHS (4, 33) have demonstrated between 7-20% refusal to participate rates, and 3-12% drop out rates for patients randomized to CPAP. We believe that we can achieve a similar rate of participation, by including regular visits and telephone contacts. Our research unit now has substantial experience in clinical trials, having participated in 18 sponsored multinational clinical trials over the past seven years, and our clinical trials staff have expertise in both patient recruitment and prevention of subject drop out. We will make use of that expertise to support this project. Our total sample size has incorporated the risk of subject drop out, and the sample is large enough so that this loss will not affect the statistical power to determine regression of carotid IMT. If the drop out rates are higher than predicted then this may induce a bias into interpretation of our results. However, we will record and understand the characteristics of all our subjects who withdraw, so that it is possible to measure and adjust for any bias. We will monitor this closely during the study and make adjustments to our recruitment strategy as required to minimize this bias as much as possible.

Statistical Analysis: *The primary outcome analysis will be performed at 6 and 12 months for regression of the early signs of atherosclerosis (reduction in IMT).* Secondary outcome variables of interest will include plaque type and extent, carotid diameter and peak systolic velocity. Power calculations show that with a sample size of 64 in each group we will have 80% power to detect a difference in IMT of 0.025mm over 12 months, assuming that the common standard deviation is 0.050mm and using a two group t-test with a 0.05 two-sided significance level. Given that Drager et al. (17) demonstrated a reduction in IMT of 9% or 0.063mm in a group of 24 patients over only four months of CPAP treatment (effect size 2.5 times that estimated for our study, in only 4 months), we believe that our power calculation is quite conservative, and that our sample size will be robust. Statistical analysis will include two group t-tests and ANOVA for multiple comparisons as appropriate. Post-treatment changes will be analyzed based on both the intention-to-treat (ITT) principle with inclusion of all study patients, and a per-protocol subset which will include patients with observed outcomes. Logistic regression analysis will be performed in the control group to search for factors associated with progression of atherosclerosis.

Significance: A reduction in atherosclerosis (decrease in IMT) will be presumed to be due to CPAP therapy, as other cardiovascular risk factors will be stable over the period of the study. This study will allow us to determine if the addition of CPAP treatment to optimal standard therapy for cardiovascular risk factors will result in regression of atherosclerosis in a population of patients with heavy snoring but only mild to moderate non-hypoxic SDB, implicating heavy snoring with SDB as a pathogenic factor in atheroma formation, and implying a possible role for treatment of heavy snoring with mild SDB in the prevention of carotid atherosclerosis.

OVERALL SIGNIFICANCE: *This study may provide support for the use of nasal CPAP therapy in patients with heavy snoring and mild to moderate, non-hypoxic SDB to prevent the development of carotid artery atherosclerosis.* In addition, this study may provide supportive evidence for the

hypothesis that heavy snoring with mild to moderate SDB is an independent risk factor for the development of carotid artery atherosclerosis. Given that carotid atherosclerosis is a major risk factor for stroke, this will have important public health implications for the future management of habitual snoring with mild to moderate SDB in the prevention of carotid atherosclerosis and stroke.
