# Supplementary material for: Predictors for carotid and femoral artery intima-media thickness in a non-diabetic sleep clinic cohort
Source: PLoS One. 2021 Jun 4;16(6):e0252569. doi: 10.1371/journal.pone.0252569 (PMC8177540; doi:10.1371/journal.pone.0252569)
Supplement: S2 Protocol — (PDF) [file pone.0252569.s003.pdf]

INTRODUCTION:

This proposal focuses on the mechanisms by which snoring and obstructive sleep apnoea syndrome (OSAS) may contribute to the development of cerebral small and large vessel disease leading to stroke. We will explore the general hypothesis that epidemiologically demonstrated links between OSAS and stroke are mediated, at least partially, through development of specific stroke promoting vascular pathologies. Retinal microvasculature morphology provides a direct window on the cerebral circulation for assessing the integrity of the microvasculature, while carotid artery morphology is an established indicator of stroke risk from macrovascular causes. We will utilise the accessibility of carotid and retinal blood vessels to test the hypothesis that:

Untreated heavy snoring and OSAS is associated with increased prevalence of cerebral microvascular disease (reflected via retinal vessel morphology and micro-emboli) and/or macrovascular disease (reflected via carotid intima-media thickness [IMT]).

The pathological mechanisms that lead to stroke in OSAS are unclear, but may include: 1) the development of cerebral microvascular disease (associated with lacunar stroke); 2) the development of carotid atherosclerosis (associated with thrombotic stroke); 3) cerebral embolic stroke from active carotid plaque disease (possibly due to snoring related carotid artery vibration); and 4) haemorrhagic stroke. Of these potential mechanisms, only the development of carotid atherosclerosis in snoring and OSAS has been investigated, with no studies exploring possible links to cerebral embolic disease and the development of cerebral microvascular disease.

Our specific hypotheses are:
1. Patients with untreated heavy snoring and OSAS will demonstrate an increased prevalence of retinal microvascular and carotid macrovascular abnormalities.
2. Microvascular and macrovascular abnormalities will increase over a night of sleep, with the overnight increase correlating with the severity of the snoring and OSAS.
3. Patients with untreated heavy snoring and OSAS, and with established carotid atherosclerotic plaque, will demonstrate an increased incidence of overnight retinal arteriolar emboli (compared with normal controls), consistent with active carotid plaque disease.
4. Early changes of cerebral microvascular disease (indicated by retinal abnormalities) and macrovascular disease (indicated by carotid atherosclerosis and IMT) present in patients with severe OSAS will regress following 12 months treatment with nasal CPAP therapy.

Project 1: ***In a cohort of 390 patients (aged >45years) with snoring and OSAS,*** we will: 1) use laboratory polysomnography (PSG) with monitoring of snoring sounds to categorise OSAS severity; 2) take retinal photographs before (pm) and after (am) sleep to quantify retinal microvascular lesions including incident retinal emboli; 3) quantify carotid artery IMT, atheromatous plaque and lumen diameter using Doppler ultrasound; 4) quantify cardiovascular risk factors before and after sleep. In untreated snoring and OSAS, Project 1 will establish: (i) the prevalence of retinal macrovascular and microvascular abnormalities (including retinal emboli); and (ii) the association between vascular abnormalities and disease severity.

Project 2: ***In a subgroup of 80 heavy snoring and OSAS patients, we will undertake a 12 month clinical trial of CPAP therapy***, following stabilisation of optimal standard therapy for cardiovascular risk factors. We will record diagnostic PSG with retinal photography (pm and am) and carotid artery ultrasound (pm) as a baseline. After institution of CPAP therapy, compliance will be monitored monthly, with repeat retinal photography (pm and am) and carotid artery ultrasound following 6 and 12 months of therapy. This study will establish if the addition of CPAP therapy will result in regression of cerebral microvascular disease (retinal abnormalities) and macrovascular disease (carotid atherosclerosis and IMT) present in patients with heavy snoring and OSAS.

To undertake this project we have an established and experienced CI/AI multidisciplinary research team incorporating expertise in clinical sleep medicine and physiology [CIA/CID/CIE],

clinical trials and stroke (CIB), retinal photography and cerebral microvascular disease (CIC and AIs Mitchell and Wong) and carotid ultrasonography (AI Larcos). Sleep studies will be undertaken in the sleep laboratory research facilities at Westmead Hospital (CIA/CID/CIE), and the retinal photography analysis will be performed in the Centre for Vision Research (CIC/AI Mitchell). Long term cohort follow-up will be co-ordinated by the George Institute, University of Sydney (CIB).

BACKGROUND:

Snoring and OSAS (sleep disordered breathing [SDB]) are a common consequence of increased upper airway resistance during sleep (3). Habitual snoring (every night or almost every night) without overt OSAS is highly prevalent in the community, occurring in approximately 40% of men and 20% of women (5, 65). The prevalence of OSAS in middle age is about 4% for men and 2% for women (65). Increasingly, it is recognized that snoring and OSAS pose real risks to health (21). Emerging data are highly suggestive of an independent role in the pathogenesis of stroke (28), hypertension (9), coronary (30) and carotid atherosclerosis (29). However, the mechanisms underlying this observed stroke risk are unknown with a wide variety of suggested possibilities. Understanding the mechanisms of this increased risk will be an important step to reduce the burden of this chronic disease arising in an ageing population with an increasing prevalence of obesity.

OSAS is now generally regarded as an independent risk factor for stroke (2, 37). Epidemiological data suggest a strong relationship between OSAS and acute cerebrovascular events. The prevalence of SDB in patients with acute stroke ranges 44-72%, and after the acute phase still remains higher than in the general population (64, 56). However, uncertainties on the role of OSAS in stroke pathogenesis relate to the observation that OSAS can both precede or follow stroke occurrence (19).

In a cross-sectional analysis of >6000 subjects from the Sleep Heart Health Study, the prevalence of stroke was 60% higher (odds ratio [OR] 1.58) among those subjects with OSAS with an apnoea-hypopnoea index (AHI) >11 events/hour (50). OSAS with an AHI of >20 /hour was associated with a 4-fold increased risk (OR 4.3) of suffering a first-ever stroke over a 4 year follow-up (2).

Pathogenic involvement of OSAS in cerebrovascular disease is suggested by the direct relationship found between the severity of nocturnal oxygen desaturation and carotid intima-media thickness (IMT) and/or the occurrence of atherosclerotic plaques in the carotid arteries of OSAS patients, independent of hypertension (4, 54). Recently, two studies in patients with untreated PSG verified severe OSAS (14, 41), at low risk from the classical cardiovascular risk factors, have demonstrated the presence of early carotid atherosclerosis (increase in IMT). Thus, patients with severe OSAS appear more likely to develop early changes of cerebral macrovascular disease.

We recently published a large cross-sectional study* (29) *in 110 heavy snorers (with only mild OSAS), with adequate power to assess associations between snoring and carotid vascular disease. The prevalence of carotid atherosclerotic plaque was 31%, with significant risk factors including age, male gender, hypertension, smoking history and heavy snoring. **The adjusted odds ratio for carotid atherosclerosis in the heavy snoring group (snoring greater than 50% sleep time) was substantially increased at 10.5 (2.1 – 51.8, 95% CI),** and was clearly the strongest association. Importantly, this study is the first to demonstrate that snoring with mild OSAS (without nocturnal hypoxia) is a strong independent risk factor for carotid atherosclerosis. In support of this, the prevalence of carotid plaque in this study population was 1.8 times greater than that reported for the general population (27), and even higher than that seen in OSAS populations (41).

The novelty and importance of this study by CIA and his team were recognised by the publication of two separate editorial commentaries in the journal *Sleep* (16, 36), both of which concluded that the relationship between snoring and carotid atherosclerosis is an exciting and important new area of research to elucidate factors contributing to cardiovascular disease risk profiles. They noted that the significance of the finding may elevate the importance of snoring from a social annoyance to a vascular risk factor. If the independent association between snoring and carotid atherosclerosis

observed in this study is indeed causal, the significantly increased risk of carotid atherosclerosis in heavy snorers, coupled with the high prevalence of snoring and mild OSAS in the community, has substantial public health implications for the prevention of stroke and cardiovascular disease.

Mechanisms that have been implicated in the increased risk of stroke in OSAS include blood pressure swings, reduction in cerebral blood flow, altered cerebral autoregulation, impaired endothelial function, accelerated atherogenesis, and prothrombotic and proinflammatory states (52). In broad terms, the occurrence of stroke may be associated with the following types of cerebral vascular disease: 1) cerebral microvascular disease (associated with lacunar stroke); 2) carotid atherosclerosis (associated with thrombotic stroke); 3) cerebral embolism from carotid plaque rupture (possibly due to carotid artery vibration) or cardiac thrombus dislodgement; and 4) cerebral haemorrhage. Of these potential mechanisms, only the prevalence of carotid atherosclerosis has been investigated, with no studies exploring the possible links of snoring and OSAS to carotid plaque embolism or the development of cerebral microvascular disease. One focus of this proposal is the role of both snoring and OSAS as causes of cerebral micro-emboli and microvascular disease, both important stroke mechanisms, that have not been previously investigated in these populations.

Retinal microvascular disease can now be readily quantified with digital retinal photography, and the qualitative and quantitative analysis of the retinal microvasculature has emerged as a powerful non-invasive tool for assessment of the cerebral microcirculation (53). Retinal photography has been revolutionised by advances in imaging and computer techniques that permit retinal microvascular signs to be non-invasively studied in great detail with computerised image processing techniques (62). High quality digital photographs of the retina can be obtained using a retinal camera. In recent years, reliable assessment methods have been developed and refined leading to a battery of qualitative and quantitative methods for evaluating retinal pathology (53).

Assessment of retinal vascular morphology is useful in two broad areas: providing clues to pathophysiological mechanisms and in risk stratification. The value in understanding pathophysiological mechanisms lies in the ability to directly view the microcirculation, and the similarities of the retinal to the cerebral circulation. There is a common embryological origin, a similar anatomical structure (non-anastomotic end-arteries) and both have a barrier to the blood (blood/brain; blood/retina barrier) (60, 62). In addition, many retinal signs have been found to have prognostic significance for stroke over and above recognised risk factors (32, 62).

Qualitative assessment of retinal vascular morphology includes: focal arteriolar narrowing, arteriovenous (AV) nicking, arteriolar wall opacity, retinopathy signs (retinal haemorrhages, microaneurysms and/or cotton wool spots), retinal emboli and retinal vein occlusion. Retinal emboli are intravascular lesions seen either along the course of retinal arterioles or at a bifurcation (42).

Quantitative assessment of retinal vascular morphology includes estimates of the internal calibre of retinal arterioles, venules and the arteriole-to-venule ratio (AVR). Recent large population-based studies have demonstrated that relative narrowing of retinal arteriolar calibre, or widening of retinal venular calibre, carry predictive significance for stroke (38, 39).

Data from a number of large population-based cohorts have provided new insights into the prognostic value of retinal microvascular signs (1, 17, 22, 25, 26, 43, 48, 58, 59, 63). Thus, in total, there are now population-based data on the prognostic value of retinal signs in more than 30,000 people (62). Data from the ARIC study reported that individuals with a smaller AVR tend to have more white matter lesions (61), MRI cerebral infarcts (10) and an increased risk of incident clinical stroke (59). Prospective data from both the Rotterdam and Cardiovascular Health studies have consistently demonstrated that larger retinal venular calibre is associated with an increased clinical risk of stroke (25, 63). The Rotterdam study has further shown that wider venular calibre is associated with cerebral infarction (25), MRI-defined white matter lesions, and lacunar infarction (26). Thus, there is considerable evidence supporting an association of altered retinal vascular calibre with both clinical and sub-clinical stroke (53).

In summary, both arteriolar narrowing and venular widening are associated with clinical outcomes such as stroke. Narrowed arteriolar calibre and smaller AVR predict clinical stroke, and hence, retinal vascular calibre has great potential as a reflection of the cerebral vasculature to predict the risk of stroke (53). In this proposal, retinal photography will help to characterise the pathophysiology of the cerebral small vessel disease related to OSAS, identify whether retinal microvascular signs are prevalent in OSAS, and whether successful treatment with CPAP leads to reversal of retinal small vessel abnormalities with presumed reduction in stroke risk.

Retinal microvascular imaging has three potential roles in snoring and OSAS patients:

1) Exploring pathophysiological mechanisms of stroke. OSAS and stroke share several classical vascular risk factors but the exact mechanism(s) of snoring and OSAS associated stroke is not known. Potential mechanisms include accelerated small vessel disease, perhaps due to hypoxia or sympathetic blood pressure surges, but could also include physical factors such as snoring vibration induced/promoted atherosclerosis (20, 29, CIA18). Retinal microvascular signs provide a method of exploring these potential mechanisms. For example, increasing severity of OSAS may provoke characteristic small vessel changes that have been associated with lacunar stroke (33). Recent meta-analysis has demonstrated that retinal venular dilatation is associated with incident stroke, and retinal hypoxia is one postulated cause (39). A relationship between the severity of nocturnal hypoxia in OSAS and wider retinal vein diameter may provide additional evidence of the importance of hypoxia in the development of this sign. Recent work from our retinal group has demonstrated that retinal microvascular signs are associated with microvascular (lacunar) stroke, and, if found to be highly prevalent in an OSAS population, would provide important new evidence to explain that the excess stroke risk from this disorder is partly due to small vessel disease (33). Work from our sleep medicine group (29) has been focused on the ability of snoring vibrations to directly cause or accelerate carotid atheromatous disease (macrovascular disease related to stroke). Complicated atheroma is a source of microemboli, snoring vibrations have the potential to disrupt carotid plaque (20), and retinal photography has the potential to identify any resultant emboli.
2) Stroke risk stratification. As retinal microvascular signs may be a surrogate for the cumulative impact from life-long burden of vascular risk factors (32), the prevalence of signs may differ by severity of snoring and OSAS. This would allow additional vascular risk stratification in OSAS patients, and would help target intensive risk factor management to those at greatest vascular risk.
3) Surrogate outcomes. CPAP treatment for those with severe OSAS is largely offered for symptomatic relief, and until the results of current RCTs are available, it is unknown whether this treatment can prevent stroke, myocardial infarction or cardiovascular death. Repeated retinal imaging of those with heavy snoring and OSAS, treated with CPAP, may demonstrate reversal of retinal microvascular signs. These data will improve our understanding on how snoring and OSAS confer excess vascular risk, and strengthen the indications for CPAP therapy to reduce vascular risk.

***Only one published study has addressed the issue of retinal microvascular abnormalities in OSAS* (6).** The Sleep Heart Health Study examined 2,927 subjects, but only 1% of subjects had a clinical diagnosis of OSAS. Thus the study was underpowered to detect retinal abnormalities as a function of OSAS. Nevertheless, the overall prevalence of retinopathy was increased at higher AHI values, and an increase in AHI from 0 to 10 was associated with a decrease in the arteriole-to-venule ratio (AVR). The authors concluded that further investigation was warranted.

Preliminary analysis of data from the **Blue Mountains Eye Study** (BMES; CIC) demonstrated a large increase in venular calibre for a self reported history of OSAS (224.5µm) versus non affected non-snorers (219.7 µm; p=0.06), suggestive of early microvascular disease. Data from the **Wisconsin Sleep Cohort** (n=491) have demonstrated that a higher AHI was positively associated with wider retinal venular calibre (for AHI >15 events/hour, OR 2.08 [1.03-2.16]; p<0.05), independent of other cardiovascular risk factors (personal communication). This supports an association of SDB with microvascular mediated cerebrovascular disease.

Intima-Media Thickness (IMT) and Cerebral Macrovascular (large vessel) Disease

Carotid IMT is used as an intermediate phenotype for early atherosclerosis, is quantifiable on a linear scale, and is the primary macrovascular outcome variable in this study. Because it can be measured simply and non-invasively, it is well suited to larger clinical studies as an end point to stratify risk. Ultrasonic measurements correlate well with histology (46), and increased IMT is associated with vascular risk factors (7, 8, 49) and the presence of more advanced atherosclerosis (44). ***A systematic review and meta-analysis demonstrated that carotid IMT is a strong predictor of future vascular events (34),*** with an adjusted relative risk of stroke of 1.18 (95% CI, 1.16 to 1.21) per 0.10 mm common carotid artery IMT increase. Our quantification of IMT will follow standardised measurement methodology (55). Ultrasound will be performed by two experienced sonographers under the direction of AI Larcos, thus minimising between-technician variation.

The potential for microvascular and macrovascular disease regression with removal of a stimulus has been confirmed in several studies. For example, antihypertensive treatment in 25 patients with untreated hypertension over one year was associated with a reduction in retinal arteriolar narrowing, a widening of arteriolar branch angle and an increase in arteriolar density (24). In another study of 51 hypertensive patients, 6 months of antihypertensive treatment was associated with a reduction in mean retinal arteriole diameter of 0.5 μm and a corresponding increase in AVR (47). Thus regression of retinal microvascular disease with effective treatment is a plausible outcome. In addition, there are now several clinical studies that have demonstrated reductions in carotid IMT (indicative of regression of early atherosclerosis) over a six to twelve month period with statin lipid profile altering medications (18, 35, 40). These studies have consistently demonstrated significant regression or slowing of progression of carotid IMT with therapy, and benefits were evident after as little as six to twelve months (40).

If OSAS constitutes an independent stimulus to the development of cerebral microvascular and macrovascular disease, it is plausible that removal of this stimulus with CPAP therapy may also be associated with regression of the early changes of vascular disease within the six to twelve month time frame. In support of this, a recent study by Drager et al (15) demonstrated that effective treatment of a group of 12 severe, hypoxic OSAS patients with CPAP for 4 months resulted in a significant decrease in carotid IMT of 0.063mm, compared with a small increase of 0.008mm in 12 control OSAS subjects. Overall, this represented a 9% reduction in IMT over only a four month treatment period. This study provides strong support that CPAP treatment is able to significantly reduce the severity of cerebral macrovascular disease within a reasonable timeframe. We propose to test this proposition in order to establish the benefits of CPAP treatment in terms of both cerebral microvascular and macrovascular risk reduction in patients with OSAS.

Preliminary Data: We have completed a pilot study in 28 subjects referred for investigation of SDB, with retinal photography performed before and after one night of PSG. Subject characteristics: age 55.1±11.9 years (mean±SD); BMI 34.4±7.3 kg/m^2; RDI 31.7±24.9 events/hour (range 0-102); snoring 49.4±26.2 % sleep epochs. The prevalence of retinal emboli was 10.7% (present in 3/28 subjects both pre and post sleep; all 3 with heavy snoring or severe OSAS). This represents an 8-fold increase in prevalence of asymptomatic retinal emboli compared with population data from the local BMES (prevalence 1.4%; 95% CI 1.0 to 1.8%) (42). Overnight, mean retinal arteriolar diameter decreased from 153.9±13.5 to 151.7±14.2 μm ($p<0.02$); mean venular diameter increased from 222.9±23.2 to 227.3±22.5 μm ($p<0.016$); and mean AVR fell from 0.69±0.06 to 0.67±0.06 ($p=0.001$; Fig 1). Correlation analysis (Spearman's) demonstrates significant associations between: (i) increasing snoring time and a decrease in retinal arteriolar diameter ($r = 0.42$, $p<0.03$; Fig 2); and (ii) overnight oxygen desaturation and decreasing AVR ($r=0.38$, $p<0.05$). In the subjects where RDI was <15 events/hour (control), there was no change in overnight retinal arteriolar or venular diameters ($p>0.5$). Thus, preliminary data demonstrate that there is: 1) no overnight change (sleep or circadian influence) in retinal microvasculature in control/mild OSAS subjects; 2) a clear overnight increase in venular diameter, and decrease in

arteriolar diameter and AVR in OSAS subjects; 3) a decrease in arteriolar diameter which is associated with increasing snoring time; and 4) an increased prevalence of retinal emboli in OSAS. These are the first data to demonstrate overnight change in the retinal microvasculature.

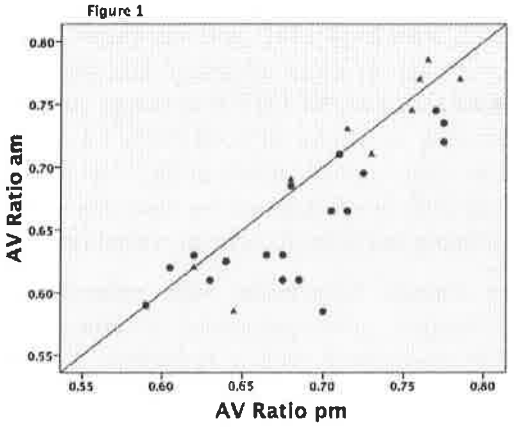

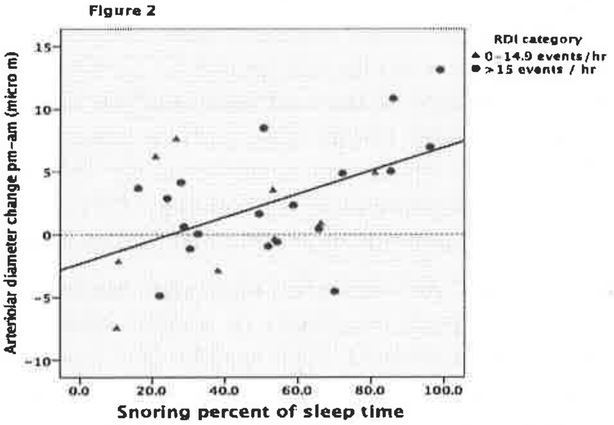

Figure 1: Identity plot of AV Ratio am versus pm. Note that controls (triangles) fall along the identity line; cases (circles) are shifted to the right, consistent with overnight decrease in AVR. Figure 2: Change in arteriolar diameter (pm-am) plotted against snoring. Note the correlation (solid line; $r=0.38$; $p<0.05$) between increasing snoring and decrease in overnight arteriolar diameter.

RESEARCH PLAN:

PROJECT 1: The Influence of Untreated Snoring and OSAS on Cerebral Vascular Disease.

Hypotheses: *1) Patients with untreated heavy snoring and OSAS will demonstrate an increased prevalence of retinal microvascular and carotid macrovascular abnormalities.*
2) Microvascular and macrovascular abnormalities will increase over a night of sleep, with the overnight increase correlating with the severity of the snoring and OSAS.
3) Patients with untreated heavy snoring and OSAS, and with established carotid atherosclerotic plaque, will demonstrate an increased incidence of overnight retinal arteriolar emboli (compared with normal controls), consistent with active carotid plaque disease.

We propose to address the above hypotheses by comparing pre and post sleep data in patients with untreated heavy snoring and OSAS and comparing any differences to those with mild or no snoring and OSAS, to directly address the following specific research questions:

1) Do patients with heavy snoring and OSAS demonstrate an increased baseline prevalence of microvascular disease (retinal vessel changes) and/or macrovascular disease (carotid artery IMT)?
2) Do patients with OSAS demonstrate a decrease in arteriolar diameter or an increase in retinal venular diameter following overnight sleep as a function of the severity of their OSAS or snoring?
3) Do patients with heavy snoring and OSAS, and with carotid atherosclerotic plaque, demonstrate a higher frequency of prevalent and overnight incident retinal arteriolar emboli?

Design: Nested case-control cohort study in snoring and OSAS referrals, comparing severe cases with control/mild cases (see below) at baseline and for overnight changes in vascular abnormalities.

Subjects: We will recruit 390 patients (130 patients/year) aged >45 years who are referred to the Westmead Hospital Sleep Laboratory for a routine diagnostic PSG for assessment of possible SDB. Exclusion criteria include past history of stroke, diabetes, atrial fibrillation, congestive cardiac failure, carotid artery surgery, and eye pathologies that preclude the measurement of retinal vessel calibre (e.g. age-related macular degeneration, glaucoma). Our Laboratory performs over 500 diagnostic PSG studies/year in this age group, with results demonstrating a Respiratory Disturbance Index (RDI) of <5 events/hr in 7% (normal controls), 5-15 events/hr in 24% (mild OSAS), 15-30 events/hr in 27% (moderate OSAS) and >30 events/hr in 42% (severe OSAS). Review of our

database indicates that 82% of target patients meet the inclusion criteria. We will stratify patient recruitment across the severity range. Allowing for a 50% participation refusal, there will be no problems recruiting target numbers.

Baseline/Anthropometry Data: We will record age, gender, body mass index, waist-hip ratio, and neck circumference. Questionnaire data will include cardiovascular risk (Framingham Questionnaire), Epworth Sleepiness Scale (ESS), snoring history, and smoking history. Detailed history of hypertension will include length of known diagnosis and current treatment. A fasting blood sample will be collected for lipid profile and glucose level.

Procedures: *PSG:* Sleep will be monitored using standard PSG proceedures for our laboratory (29). In addition, we will record snoring sounds using a room sound level meter and quantify snore-related tissue vibration energy using an accelerometer attached to the skin surface of the neck.
Carotid Artery Ultrasound: Doppler ultrasound examination of both carotid arteries will be used to measure IMT (57) of the common carotid, plaque type and extent, lumen diameter, and peak systolic velocity.
Retinal Photography: Each eye will be photographed following a standardized protocol using a high-resolution digital camera (Canon non-mydriatic). Two colour retinal photographs will be taken from each eye, one centred on the optic disc (Diabetic Retinopathy Study [DRS] standard field 1) and another on the macula (DRS standard field 2) (13). Digital images will be graded for retinal microvascular lesions using standard protocols under direction of CIC. Qualitative signs will be recorded (see Background p3). The intra-grader reliability for detecting focal arteriolar narrowing and AV nicking is (kappa statistic) 0.80 and 0.87, respectively (57).
A computer-assisted grading method with high reproducibility will be used to measure the retinal vessel diameters and arteriole-to-venule ratio (AVR). Average retinal arteriolar or venular width (diameter) is calculated using the Parr-Hubbard formula (23, 45) and presented as the central retinal arteriolar equivalent (CRAE) or central retinal venular equivalent (CRVE). AVR can then be calculated from CRAE and CRVE. Intra- and inter-grader reliability of this method is high (51). Retinal emboli are intravascular lesions seen along the course of retinal arterioles, and may be present at a bifurcation. Detailed methods of grading retinal abnormalities have been described (57).

Protocol: Subjects will undergo an overnight PSG study. Carotid ultrasound, retinal photography and supine blood pressure will be performed at ~2000hrs and repeated at ~0600hrs. A major outcome focuses on overnight change in measurements, where subjects act as their own controls.

Statistical Analysis and Sample Size: Univariate relationships between measures of OSAS or snoring severity (AHI, Arousal Index, Oxygen Desaturation Index, snore index) [independent variables], and overnight change in CRAE, CRVE, AVR and IMT [dependent variables], will be examined using linear regression analysis for continuous data and X^2 analysis for categorical data. Stepwise multiple linear regression or logistic regression will be used to examine associations of OSAS and snoring (continuous data) with dependent variables, including any interactions with anthropometric and vascular risk factor data. From our preliminary data, a sample size of 384 for the cohort study will have 80% power to detect a difference in the CRVE means of 8.0 μm (and difference in CRAE means of 13.6 μm), assuming that the common standard deviation is 24.1 μm for CRVE and 13.6 μm for CRAE, using a two group t-test with two sided significance of p=0.017. For the overnight changes, a sample size of 146 will have 80% power to detect a correlation co-efficient of 0.25 between AVR and snoring using linear regression, assuming normal distribution of AVR and a two sided significance level of p=0.025. Power estimates are conservative based on the dependent variables with the smallest detected signals from our preliminary retinal data.

Outcomes: This study will establish the relationship between snoring / OSAS and the presence of cerebral microvascular and macrovascular disease in a cross section of subjects. Overnight changes in the retinal vessel calibre will demonstrate for the first time a mechanism by which SDB acutely and adversely affects the microvasculature. In addition, if the cross-sectional analysis of subjects

demonstrates the same changes in vessels as a function of SDB severity, then this would support these changes becoming chronic over time. Finally, we will examine for the presence of retinal emboli before and after sleep, which will provide the first evidence for snoring and OSAS being associated with active plaque disease, with potential for plaque disruption and embolic stroke.

PROJECT 2: Regression of Cerebral Vascular Disease in Severe OSAS using CPAP Therapy

Hypothesis: *Early changes of cerebral microvascular disease (indicated by retinal abnormalities) and macrovascular disease (indicated by carotid atherosclerosis and IMT) present in patients with severe OSAS will regress following 12 months treatment with nasal CPAP therapy.*

Design: This will be a one-year prospective, longitudinal, observational study of OSAS patients (acting as their own controls). Following discussion with our Ethics Committee, it would not be acceptable to have a randomised, controlled design with a sham treatment arm over a one year period for patients with severe, symptomatic OSAS.

Subjects: We will recruit 80 patients from Project 1 (40 in year 1 and 40 in year 2), who have been diagnosed with severe, symptomatic OSAS (RDI >30 events/hr, ESS >9). Exclusion criteria are as for Project 1, plus current smoking or an inability to tolerate CPAP therapy. Successive patients from Project 1 who meet the inclusion criteria will be approached to participate. Over the first two years, our laboratory will diagnose over 400 patients who meet the inclusion criteria.

Baseline/Anthropometry Data: Same as for Project 1.
Procedures: PSG, Carotid Artery Ultrasound, Retinal Photography as for Project 1.

Protocol: Recruited subjects (n=40/year for first 2 years) will complete a run-in period, with review of clinical history, blood lipids, and blood pressure to ensure that subjects have been stabilised on optimal standard therapy for cardiovascular risk factors (normal values with no clinically significant changes). Optimal standard therapy will include continued non-smoking, maintenance of weight, medication (as required) to keep daytime blood pressure and blood lipid profile normal. When clinically stable, diagnostic PSG with repeat carotid Doppler ultrasound (pm) and retinal photography (pm and am) will be performed as a baseline. Subjects will then have a laboratory CPAP titration study, to eliminate snoring and obstructive events as per laboratory protocol.
The study treatment period will run for 12 months, with monthly monitoring of CPAP compliance (machine hours at pressure recorded), regular study co-ordinator follow up, and continued monitoring and treatment of cardiovascular risk factors. Subjects will be stratified during analysis according to medication use for cardiovascular risk factors and CPAP compliance.
Overnight CPAP review studies using the patient's CPAP treatment pressure will be undertaken at 6 months and one year. Repeat carotid Doppler ultrasound (pm), retinal photography (pm and am), blood pressure (pm and am), fasting blood lipids and sugar, and weight will be measured.
This duration of study is adequate to detect clinically relevant outcomes. An increase in IMT of >0.03 mm/year is detectable and clinically relevant, as it is associated with a doubling in incidence of coronary events (44). We anticipate a reduction of at least this amount (15). For retinal photography, a decrease in 3.5 μm/year of arteriolar calibre is detectable, measurable in a one year time frame, and associated with a 10 mmHg increase in mean arterial blood pressure (31), making it clinically relevant. Both arteriolar narrowing and venular widening are predictive of stroke (59).

We have chosen to compare the same subjects before vs after the intervention but not to have an untreated control arm in this study due to efficiency of paired comparison and the well recognised ethical problems with not treating severe OSAS. This will enable a valid paired comparison with the differences between the before and after treatment measures being related to the treatment intervention over 1 year. The primary outcome variables (CRAE, CRVE, AVR and IMT) are objective measures of structural changes which are unlikely to spontaneously improve without intervention, or be affected by a placebo type response.

We acknowledge that compliance with CPAP treatment will be a critical factor to the success of this trial. Patients will be offered as much support as required in the initial phases of the trial by the study co-ordinator, with four weekly review throughout the study. Patients will be able to contact the study co-ordinator at any time to resolve problems. CPAP compliance will be monitored objectively by nights and hours of use from machine downloads at study visits, together with compliance with routine medication(s). We will target a minimum compliance of 4 hours per night.

Statistical Analysis and Sample Size: Power analysis is based on data relating to the observed reduction in IMT during CPAP therapy (15). Power calculations show that with a **sample size of 64** we will have 80% power to detect a difference in IMT of 0.025 mm over 12 months, assuming that the common standard deviation is 0.050 mm and using a two group t-test with a 0.05 two-sided significance level. Given the demonstrated reduction in IMT of 9% (0.063 mm) in 24 patients over only four months of CPAP treatment (15), we believe that our power calculation is quite conservative, and that our sample size is robust. Similarly, following 12 months of treatment for hypertension, there have been demonstrated improvements in retinal vessel diameter of 12.5% (24), consistent with the changes in IMT. We will study 80 subjects to allow for a 20% drop-out rate.

Significance: A reduction in atherosclerosis (decrease in IMT) and retinal microvascular changes (increase in CRAE, decrease in CRVE) will be related to CPAP therapy, as other cardiovascular risk factors will be stable over the study period, and any other functional changes will be adjusted for during analysis. ***This study will determine if CPAP treatment in patients with severe OSAS results in regression of both cerebral microvascular and macrovascular disease***, implicating OSAS as a pathogenic factor in cerebral vascular disease, and implying a possible role for treatment of OSAS in risk reduction from cerebral vascular disease.

FUTURE DIRECTIONS: Long-term risk of stroke in Heavy Snoring and OSAS Population

Hypothesis: *Long-term lifetime follow-up of a cohort of well-described patients with heavy snoring and OSAS will confirm the excess risk of stroke, and the predominant stroke subtypes.*

We intend to follow patients recruited in this project over their lifetime. Consent for lifetime follow-up for mortality of the subjects recruited in these projects will allow us to determine the risk of stroke. We will obtain date and cause of death via data linkage with the Australian National Death Index, and also obtain subsequent stroke events from the Centre for Health Record Linkage (CHeReL), ***which will not require additional NHMRC funding.*** Our current national follow-up for participants in RCTs (12) allows a cost-effective system of collecting events. This type of follow-up is inexpensive and can be sustained over time by CIA/CIB. There would be a pre-specified plan to continue follow-up at regular future intervals.

OUTCOMES AND SIGNIFICANCE:

These two projects investigate the macrovascular and microvascular mechanisms of the associations between both heavy snoring and OSAS with an increased risk of stroke. The use of retinal photography (for microvascular disease) and carotid ultrasound IMT (for macrovascular disease) are exciting new investigations which may provide practical clinical tools for stratification of overall cardiovascular risk in snoring and OSAS subjects. In addition, the second study will provide evidence supporting the benefit of CPAP treatment in reduction of cerebral microvascular and macrovascular disease, with consequent stroke risk reduction. Given the high prevalence of snoring and OSAS in the increasingly obese middle to older aged population, findings from these studies will demonstrate a new target for interventions to lower cardiovascular disease risk profiles in the population, and to further reduce the disease burden and premature deaths from stroke and other cardiovascular events.

REFERENCES

1. ARIC investigators. The Atherosclerosis Risk in Communities (ARIC) Study: design and objectives. Am J Epidemiol. 129:687-702, 1989.
2. Artz M *et al.* Association of SDB and occurrence of stroke. *AJRCCM.* 172: 1447-1451, 2005.
3. Ayappa I *et al.* The upper airway in sleep. *Sleep Med. Rev.* 7(1):9-33, 2003.
4. Baguet JP *et al.* The severity of oxygen desaturation is predictive of carotid wall thickening and plaque occurrence. *Chest.* 128: 3407-3412, 2005.
5. Bearpark H *et al.* Snoring and sleep apnoea. *AJRCCM.* 151(5): 1459-1465, 1995.
6. Boland LL *et al.* Sleep disordered breathing is not associated with the presence of retinal microvascular abnormalities: The Sleep Health Heart Study. *Sleep.* 27: 467-73, 2004.
7. Bots ML *et al.* Common carotid IMT and risk of stroke and myocardial infarction: the Rotterdam Study. *Circulation.* 96:1432-1437, 1997.
8. Chambless LE *et al.* Carotid wall thickness is predictive of incident clinical stroke: The Atherosclerosis Risks in Communities (ARIC) Study. *Am. J. Epidemiol.* 151:478-487, 2000.
9. Cho N *et al.* Relation of habitual snoring with components of metabolic syndrome in Korean adults. *Diabetes Res. Clin. Pract.* 71(3): 256-263, 2006.
10. Cooper LS *et al.* Retinal microvascular abnormalities and MRI-defined subclinical cerebral infarction: the Atherosclerosis Risk in Communities Study. *Stroke.* 37: 82-86, 2006.
11. Cugati S, **Wang JJ**, Rochtchina E, **Mitchell P**. 10-Year Incidence of retinal emboli in an Older Population. *Stroke.* 37: 908-910, 2006.
12. Dennis M, **Lindley R,** *et al.* Effectiveness of thigh-length graduated compression stockings to reduce the risk of deep vein thrombosis after stroke (CLOTS trial 1). *Lancet.* 373: 1958-1965, 2009.
13. Diabetic Retinopathy Study Research Group. DRS Report Number7: A modification of the Airlie House classification of diabetic retinopathy. *Investigative Ophthalmology & Visual Science.* 21: 210, 1981.
14. Drager LF *et al.* Early signs of atherosclerosis in OSA. *AJRCCM.* 172(5): 613-618, 2005.
15. Drager LF *et al.* Effects of CPAP on early signs of atherosclerosis in OSA. *AJRCCM* 176: 706-712, 2007.
16. Drager LF *et al.* Heavy snoring and carotid atherosclerosis: is there more than an association? *Sleep* 31(10): 1335, 2008.
17. Fried LP, Borhani NO, Enright P, *et al.* The Cardiovascular Health Study: design and rationale. *Ann Epidemiol.* 1: 263-76, 1991.
18. Furberg CD, et al. Effect of lovastatin on early carotid atherosclerosis and cardiovascular events. *Circulation.* 90(4):1679-1687, 1994.
19. Gibson GJ. Sleep Disordered Breathing and the outcome of stroke. *Thorax.* 59: 361-363, 2004.
20. Hedner J *et al.* Speculations on the interaction between vascular disease and OSA. Sleep and Breathing. Edited: Saunders NA and Sullivan C, New York: Dekker 1994: 823-846.
21. Hoffstein V. Is snoring dangerous to your health? *Sleep.* 19 (6):506-516. 1996.
22. Hofman A, Grobbee DE, de Jong PT, van den Ouweland FA. Determinants of disease and disability in the elderly: the Rotterdam Elderly Study. *Eur J Epidemiol.* 7: 403-22, 1991.
23. Hubbard LD, *et al.* Methods for evaluation of retinal microvascular abnormalities associated with hypertension/sclerosis in the Atherosclerosis Risk in Communities Study. *Ophthalmology.* 106: 2269-2280, 1999.
24. Hughes AD *et al.* Effect of antihypertensive treatment on retinal microvascular changes in hypertension. *Journal of Hypertension.* 26: 1703-07, 2008.
25. Ikram MK *et al.* Retinal Vessel diameters and risk of stroke: the Rotterdam Study. *Neurology.* 66: 1339-43, 2006.
26. Ikram MK *et al.* Retinal vessel diameters and cerebral small vessel disease: the Rotterdam Scan Study. *Brain.* 129: 182-8, 2006.

27. Irace C *et al.* Wall shear stress is associated with intima-media thickness and carotid atherosclerosis in subjects at low coronary heart disease risk. *Stroke.* 35(2): 464-468, 2004.
28. Koskenvuo M *et al.* Snoring as a risk factor for IHD and stroke in men. *Br. Med. J. (Clin. Res. Ed.).*294: 643, 1987.
29. Lee SA, Amis TC, Byth K, Larcos G, Kairaitis K, Robinson TD, **Wheatley JR**: Heavy snoring as a cause of carotid artery atherosclerosis. *Sleep.* 31:1207-1213, 2008.
30. Leineweber C *et al.* Snoring and progression of coronary artery disease. *Sleep.* 27(7):1344-1349, 2004.
31. Leung H, **Wang JJ, Mitchell P** *et al.* Relationships between age, blood pressure, and retinal vessel diameters in an older population. *Invest Opthalmol Vis Sci.* 44: 2900-04, 2003.
32. Liew G, **Wang JJ, Mitchell P,** Wong TY. Retinal vascular imaging: A new tool in microvascular disease research. *Circulation Cardiovascular Imaging.* 1: 156-161, 2008.
33. **Lindley RI, Wang JJ, Mitchell P** *et al.* Retinal microvasculature in acute lacunar stroke: a cross-sectional study. *Lancet Neurology.* 8: 628-34, 2009.
34. Lorenz MW *et al.* Carotid IMT indicates a higher vascular across a wide age range: prospective data from the Carotid Atherosclerosis Progression Study. *Stroke.* 37: 87-92, 2006.
35. MacMahon S, et al. Effects of lowering average of below-average cholesterol levels on the progression of carotid atherosclerosis. *Circulation.* 97(18):1784-1790, 1998.
36. Malhotra *et al.* Not so good vibrations. *Sleep.* 31(9):1207-1213, 2008.
37. Marin JM *et al.* Long-term cardiovascular outcomes in men with OSAH with or without treatment with continuous positive airway pressure. *Lancet.* 365(9464): 1046-1053, 2005.
38. McGeechan K, **Wang JJ, Mitchell P**, *et al.* Retinal vessel caliber and prediction of coronary heart disease: A systematic review and meta-analysis. *Annals of Internal Medicine.* 151:404-413, 2009.
39. McGeechan K, **Wang JJ, Mitchell P**, *et al.* Prediction of incident stroke events based on retinal vessel caliber: A systematic review and individual participant meta-analysis. *American Journal of Epidemiology.* 170:1323-1332, 2009.
40. Mercuri M, et al. Pravastatin reduces carotid IMT progression in an asymptomatic hypercholesterolemic Mediterranean population. *Am. J. Med.* 101(6):627-634, 1996.
41. Minoguchi K *et al.* Increased carotid IMT in OSA. *AJRCCM.* 172: 625-30, 2005.
42. **Mitchell P, Wang JJ**, Li W, Leeder SR, Smith W. Prevalence of asymptomatic retinal emboli. *Stroke.* 28: 63-66, 1997.
43. **Mitchell P, Wang JJ,** Wong TY, Smith W, Klein R, Leeder SR. Retinal microvascular signs and risk of stroke and stroke mortality. *Neurology.* 65: 1005-9, 2005.
44. O'Leary DH *et al.* Carotid-artery IMT as a risk factor for MI and stroke in older adults. Cardiovascular Health Study Collaborative Research Group. *N. Eng. J. Med.* 340:14-22, 1999.
45. Parr JC, Spears GF. General caliber of the retinal arteries expressed as the equivalent width of the central retinal artery. *Am J Ophthalmol.* 77: 472-477, 1974.
46. Pignoli P *et al.* Intimal plus medial thickness of the arterial wall: a direct measurement with ultrasound imaging. *Circulation.* 74:1399-1406, 1986.
47. Pose-Reino A *et al.* Regression of Alterations in Retinal Microcirculation Following Treatment for Arterial Hypertension. *J Clin Hypertens.* 8: 590-95, 2006.
48. Rosamond WD, *et al.* Stroke incidence and survival among middle-aged adults: 9-year follow-up of the Atherosclerosis Risk in Communities (ARIC) cohort. *Stroke.* 30: 736-43, 1999.
49. Rosvall M *et al.* Incidence of stroke is related to carotid IMT even in the absence of plaque. *Atherosclerosis.* 179: 235-331, 2005.
50. Shahar E et al. Sleep-disordered breathing and cardiovascular disease: cross-sectional results of the Sleep Heart Health Study. Am J Resp Crit Care Med. 163: 19-25, 2001.
51. Sherry LM, **Wang JJ**, *et al.* Reliability of computer-assisted retinal vessel measurement in a population. *Clinical & Experimental Ophthalmology.* 30: 179-82, 2002.

52. Somers VK *et al.* AHA/ACC Sleep Apnea and Cardiovascular Disease. *Journal of the American College of Cardiology.* 52: 686-717, 2008.
53. Sun C, **Wang JJ** *et al.* Retinal Vascular Caliber: Systematic, Environmental, and Genetic Associations. *Survey of Ophthalmology.* 54: 74-95, 2009.
54. Suzuki T *et al.* Obstructive sleep apnea and carotid artery intima-media thickness. *Sleep.* 27: 129-133, 2004.
55. Touboul PJ *et al.* Mannheim carotid IMT consensus. *Cerebrovasc. Dis.* 23:75-80, 2007.
56. Turkington PM *et al.* Sleep Disordered Breathing following stroke. *Monaldi Arch Chest Dis.* 61: 167-161, 2004.
57. **Wang JJ, Mitchell P** *et al.* Hypertensive retinal vessel wall signs in a general older population: the Blue Mountains Eye Study. *Hypertension.* 42: 534-41, 2003.
58. **Wang JJ,** Liew G, Klein R, *et al.* Retinal vessel diameter and cardiovascular mortality: pooled data analysis from two older populations. *Eur Heart J.* 28: 1984-92, 2007.
59. Wong TY *et al.* Retinal microvascular abnormalities and incident stroke: the Atherosclerosis Risk in Communities Study. *Lancet.* 358: 1334-40, 2001.
60. Wong TY *et al.* Retinal microvascular abnormalities and their relationship with hypertension, cardiovascular disease, and mortality. *Surv Ophthalmology.* 46: 59-80, 2001.
61. Wong TY *et al.* Cerebral white matter lesions, retinopathy, and incident clinical stroke. *JAMA.* 288: 67-74, 2002.
62. Wong TY. Is retinal photography useful in the measurement of stroke risk? *Lancet Neurol.* 3: 179-183, 2004.
63. Wong TY *et al.* Quantitative retinal venular caliber and risk of cardiovascular disease in older persons: the cardiovascular health study. *Arch Intern Med.* 166: 2388-94, 2006.
64. Yaggi H *et al.* OSA and stroke. *Lancet Neurology.* 3(6): 333-342, 2004.
65. Young T *et al.* Occurrence of SDB in middle-aged adults. *NEJM.* 328(17):1230-1235, 1993.
